# Re-energizing health literacy in Wales: a testbed for health, education and prosperity for all

**DOI:** 10.1093/heapro/daae055

**Published:** 2024-06-12

**Authors:** Emily Marchant

**Affiliations:** Department of Education and Childhood Studies, Singleton Park Campus, Swansea University, SA2 8PP, UK

**Keywords:** health literacy, children and young people, schools, curriculum, curricula, health and well-being, policy, strategy

## Abstract

A growing body of evidence demonstrates the importance of enhancing health literacy for improved health outcomes, self-reported health, lower health services use and disease prevention. Importantly, improving health literacy has great potential to reduce health inequities and inequalities. The World Health Organization (WHO) has identified health literacy as a global priority, viewing it as a right and a fundamental competency necessary to function within modern society. Building health literacy foundations should begin in early childhood, including focus within educational frameworks and school curricula. The WHO advocate for governments to embed it as an explicit goal. In response, it has received significant international policy and strategy focus, in addition to the development of country-level action plans. In Wales, UK, it was identified as a priority in 2010, but despite wider developments spanning health and social care, well-being, economy and education policy, growth in health literacy has stalled since. Optimizing health literacy would act as an indirect enabler to a range of Welsh policies and strategies. A promising avenue for strengthening the health literacy of current and future generations is through ongoing significant national education reforms and the introduction of the new *Curriculum for Wales*. One of four overarching purposes of this curriculum is *healthy, confident individuals*, and *health and well-being* constitutes one of six statutory curriculum areas. Tracking the impact of this on children and young people’s health literacy offers opportunities for Wales to model and gain traction as a national-scale health literacy policy testbed. This requires re-energizing health literacy as a national priority.


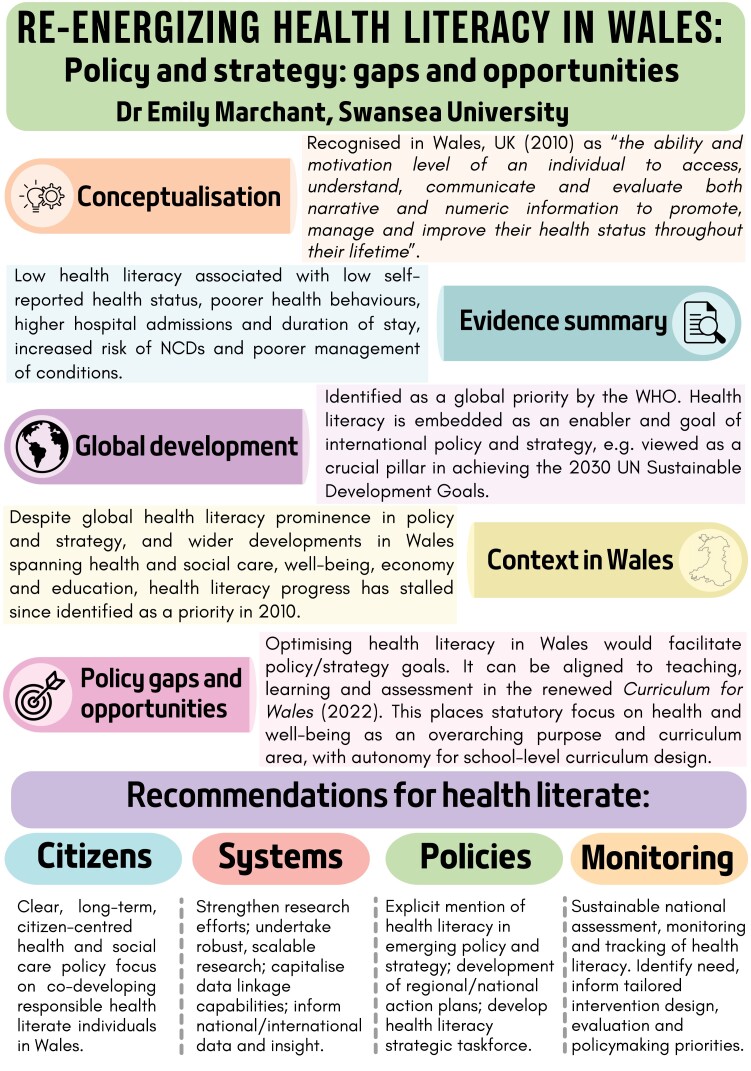

**Contribution to Health Promotion**

- Health literacy was identified as a priority in 2010 in Wales, however, progress has since stalled despite wider policy developments within health, social care, education and economy.
- For children and young people, the new *Curriculum for Wales* (from September 2022) provides opportunities to enhance health literacy, with a statutory focus on health and well-being as a curriculum area and overarching purpose.
- It is essential to re-energize health literacy as a national priority in Wales; this includes embedding as an explicit concept and goal within policy/strategy, the development of national action plans and enhancing monitoring and tracking tools.

## BACKGROUND AND CONCEPTUALIZATION

The field of health literacy, which gained prominence in the 1970s (Simonds, 1974), has grown exponentially over the last few decades and received significant international policy attention in recent years. Health literacy is a modifiable factor that contributes to the promotion and maintenance of health and well-being throughout an individual’s life (Gibney *et al*., 2020). Whilst multiple definitions exist globally (Sørensen *et al*., 2012), the definition adopted within Wales, UK in 2010 is ‘*the ability and motivation level of an individual to access, understand, communicate and evaluate both narrative and numeric information to promote, manage and improve their health status throughout their lifetime’* (Puntoni, 2010).

Health literacy has primarily been conceptualized through the fields of public health, health promotion, and social sciences with a focus on information literacy and decision-making. A foundational model proposed by Nutbeam in 2000 (Nutbeam, 2000) and recognized within Wales (Puntoni, 2010) comprises of three domains: *functional, interactive and critical:*

- *Functional*: basic reading, writing and numeracy skills to understand health-related information.
- *Interactive*: more advanced cognitive and literacy skills that enable an individual to interpret, extract and apply information from different forms of health communication.
- *Critical*: critically analysing and using health information to exert greater control over one’s health and life.

Childhood is a period of significant formative development where knowledge, skills and capacities impacting health are acquired and health behaviours established which can be tracked into adulthood. Building the foundations for health literacy should begin in early childhood with a focus on education settings (WHO Regional Office for Europe, 2013b). The World Health Organization (WHO) advocate embedding health literacy within educational frameworks and school curricula (World Health Organization, 2021). In Wales, children and young people have been identified as a priority group in recent policy and parliamentary reviews (Welsh Government, 2018, 2021). However, health literacy remains a neglected field within policy and strategy and there is a significant gap in understanding of children and young people’s health literacy needs in Wales. This perspective piece explores the concept of health literacy with a focus on children and young people aged 0–16 (from birth to the end of compulsory education) within the context of research, policy and strategy in Wales.

## EVIDENCE SUMMARY

Whilst data for Wales, and particularly children is lacking, a growing body of European and international evidence demonstrates the importance of health literacy on a range of health outcomes and healthcare savings costs. Between one-half and one-third of European adults have low health literacy and struggle to manage health information relevant to themselves and others in different contexts. Lower health literacy is associated with higher hospital admissions, duration of hospital stay and likelihood of readmission (Shahid *et al*., 2022), thus, is a driver of higher healthcare costs. The NHS in Wales is the Welsh Government’s largest area of expenditure (Stats Wales, 2023), and a significant cause of emergency hospital admissions is due to largely preventable non-communicable diseases (NCDs) (Robbins *et al*., 2021). As modifiable lifestyle factors increase risk including tobacco use, poor nutrition and physical inactivity (WHO, 2023b), health literacy is an important tool in the prevention and management of NCDs, which are responsible for the majority of chronic diseases and nearly three-quarters of deaths worldwide (Edwards *et al.*, 2012; WHO, 2023c). The gap in premature deaths from NCDs between the least and most deprived areas in Wales is increasing and is two and a half times greater for those in the most deprived areas (Public Health Wales, 2022). Reducing the impact of low health literacy on health services is essential and has the potential not only to large healthcare savings but also to improve population-level outcomes.

For children, childhood obesity and physical inactivity are two of the most significant risk factors for NCD prevention (NCD Risk Factor Collaboration (NCD-RisC), 2017). This is of great concern in Wales where over a quarter of children are overweight or obese (Public Health Wales NHS Trust, 2023) and almost half of children (49%) do not currently meet physical activity guidelines (Richards *et al*., 2022). Furthermore, inequalities persist in these domains for sex, ethnicity, disability and socioeconomic status. Evidence demonstrates associations between low health literacy and excess body weight, overweight/obesity and physical inactivity in children (Chrissini and Panagiotakos, 2021; Kanellopoulou *et al*., 2021). Thus, encouraging healthy behaviours, minimizing the risk of developing NCDs and reducing health inequalities are key priorities in Wales; enhancing health literacy is fundamental to this.

One of the greatest potential outcomes of improving population health literacy is reducing health inequities and inequalities, this is a key policy priority in Wales. Health literacy is unequally distributed across populations, mirroring the social gradient of wider health outcomes (Sørensen *et al*., 2015). The highest proportions of low health literacy in Europe are observed amongst the lowest socioeconomic groups, those with the lowest education and the poorest health outcomes (Sørensen *et al*., 2015). Health literacy is therefore recognized as a social determinant of health (Rowlands *et al.*, 2017). This is important in the context of Wales, where 28% of children live in poverty, defined as living in households earning 60% of the median UK household income (Bevan Foundation, 2020). This is one of the highest rates in the UK, and despite significant policy focus on inequalities in Wales, rates have remained steady over the last decade.

The importance of health literacy through individual and collective decision-making and agency was brought to the forefront during the COVID-19 pandemic (Paakkari and Okan, 2020; Abel and McQueen, 2021). Experts referred to health literacy as a ‘*social vaccine*’, empowering citizens and increasing community capacity (Okan *et al.*, 2023). Research highlighted the impacts of school closures on children’s health behaviours and well-being (James *et al.*, 2021; Marchant *et al*., 2021). For school-aged children, evidence suggests an association between children reporting healthy behaviours such as physical activity with being tested, and testing positive for COVID-19 (Marchant *et al*., 2022). These findings may reflect proxy parental health literacy, with health-literate parents more likely to access/understand/interpret/apply pandemic-related information, recognize their child’s symptoms and access community testing.

## INTERNATIONAL HEALTH LITERACY PROMINENCE

The WHO identified health literacy as a global priority, advocating that health literacy, like general literacy, should be viewed as a right and a fundamental competency necessary to function within modern society (WHO, 2023a). At an individual level, adequate health literacy provides the resource for informed decision-making. However, health literacy is not the sole responsibility of individuals. It requires the support of communities, settings and governments, and is influenced by societal and political contexts and priorities.

The WHO has published a mandate for health literacy and encourages governments to prioritize enhancing population health literacy as an explicit goal within all policies (WHO, 2023a). This is reflected by the prominence of health literacy as an enabler of international and European policy and strategy, such as a crucial pillar in achieving the 2030 UN Sustainable Development Goals. Health literacy is embedded within the European Commission’s *Together for Health* (Commission of the European Communities, 2007) regarding citizen empowerment and is a priority area in the *Health 2020: a European policy framework* (WHO Regional Office for Europe, 2013a). The development of a roadmap guides policymakers, organizations and communities in the adoption and implementation of policies or strategies on health literacy (WHO Regional Office for Europe, 2019). In response, several countries have introduced health literacy action plans including Germany, Austria, Australia and Scotland (Council of Europe, 2023), though no such health literacy investment or action plan currently exists in Wales.

## HEALTH LITERACY POLICY AND STRATEGY IN WALES: GAPS AND OPPORTUNITIES

Despite developments in health literacy prominence within European and international policy and strategy, growth in Wales has stalled since identified as a priority in 2010 (Puntoni, 2010). The author advocated for ‘*a long-term vision and commitment to measure health literacy at population level*’ and noted an absence of evidence-based interventions that can achieve the goal of enhancing health literacy for all. Despite further international focus, nothing concrete has yet been implemented.

However, there have been wider public health and education developments within Wales, and a general policy and strategy shift across a range of domains spanning health and social care, well-being, economy and education. Currently, this policy focus is framed in the context of challenging financial and budgetary environments at all levels of government and public services in Wales (Welsh Government, 2023b). Ongoing system-level financial impact of the COVID-19 pandemic on the health and social care system has been observed. Recent short-term investments in the health system by the Welsh Government appear to focus on specific priority areas to address immediate system-level pressures (Deans and Davies, 2023), with perhaps less focus on prevention, health promotion and areas that could support and develop lifelong health literacy (Stats Wales, 2023).

Key pieces of health and social care, economy and education policy and strategy in Wales are summarized below, highlighting where health literacy underpins strategy goals and visions and acts as an indirect enabler in achieving impact. Gaps in existing policy and strategy are highlighted, and opportunities for emerging policy and strategy are proposed.

### Health and social care

The *Fairer Health Outcomes for All: Reducing Inequities in Health Strategic Action Plan* (2011) is underpinned by prevention and early intervention (Health Inequalities Portal, 2023). This strategy outlines a set of actions to reduce health inequities, including *Improving health literacy,* with all action areas reflective of priorities within the Marmot Review (Marmot, 2010). However, the action plan is no longer available to view online and nothing concrete addressing health literacy as a priority has been observed since. The opportunity for an all-Wales health literacy action plan is urgently required, involving collaboration between researchers and stakeholders across public health, health promotion, education and wider.

More recently, the Gwent Public Services Board (PSB) which brings together public bodies across the South Eastern region of Wales has committed to becoming a Marmot Region (Aitken, 2022). This is largely driven as a means of achieving the goals set out in the *Well-being of Future Generations (Wales) Act 2015* (Welsh Government, 2015). The Region will adopt the Marmot principles, many of which align with the field of health literacy such as *Strengthen the role and impact of ill-health prevention*, to guide action towards reducing health inequalities and inequities.

As the first country globally to legislate on future generations, the *Well-being of Future Generations (Wales) Act 2015* (Welsh Government, 2015) is a legally binding commitment to improving the well-being of people in Wales. Two of the seven well-being goals relevant to health literacy are *A healthier Wales* and *A more equal Wales.* Progress is measured through national indicators, including *Percentage of adults/children with two or more healthy lifestyle behaviours*. This indicator is heavily reliant on health literacy, though no explicit mention of the term is included within the guidance and children are only assessed aged 11 and above. Prioritizing and optimizing the health literacy of children and young people today is in the interests of current and future generations.

*A healthier Wales* (2021) (Welsh Government, 2021) sets out a vision for ‘*keeping people healthy and well*’. It focuses on prevention and self-management, placing emphasis on supporting people to manage their own health and well-being. This advocates for greater individual responsibility regarding lifestyle factors including smoking, diet and exercise. Managing individual health and well-being requires informed decisions of actions and behaviours. Though health literacy is not addressed within the plan, it is fundamental to managing one’s health and well-being. For children and young people, it recognizes the importance of the new *Curriculum for Wales (CfW)* to build insights and knowledge development relating to lifestyle factors, especially with renewed prominence of health and well-being within the curriculum (Welsh Government, 2023a).

*The Public Health Outcomes Framework: measuring the health and well-being of a nation* (2016) (Public Health Wales, 2016) places focus on prevention, inequalities, inequities and the social determinants of health, these are all strongly correlated with health literacy. A key theme that directly aligns with the field of health literacy is *Individual responsibility—empowering and enabling people to take personal responsibility for improving their own health.* However, there lacks explicit mention of health literacy, likely due to a lack of health literacy measurement tools to base and track outcomes and indicators. Developing a national measurement of health literacy, as highlighted within the 2010 scoping review (Puntoni, 2010) would strengthen the power of assessment and tracking within the Framework.

### Economy

The Welsh Government’s 2018*Prosperity for All* (Welsh Government, 2019) economic action plan demonstrates a clear commitment to promoting health and building healthy communities. *Healthy and Active* is one of four themes underpinning this strategy, recognizing the links between building skills, employment and economic outcomes with improved health outcomes. This views health and well-being as one of the fundamental drivers and products of economic growth and prosperity for all. It recognizes the critical role of schools in embedding healthy and active behaviours and lifestyles. This can be achieved through optimizing children’s health literacy as a social determinant of health and employment through the *CfW*. On the contrary, inadequate health literacy can have significant economic implications, placing additional pressure on an already overburdened health and social care system, negatively impacting the labour market and preventing true prosperity for all.

### Education

Educational settings have been highlighted as one of three key intervention areas to target approaches enhancing school-aged children’s health literacy (Kickbusch, 2008). From an early age, knowledge, skills and capacities can be developed to empower decision-making impacting health. With education a devolved responsibility of the Welsh Government, one of the most promising avenues for strengthening the health literacy of current and future generations is through ongoing significant national education reforms. The *Curriculum for Wales* (*CfW)* for learners aged 3–16 rolled out across Wales from September 2022 (Welsh Government, 2023a).

The wider curriculum vision is underpinned by *Four Purposes;* ‘*the starting point and aspiration for every child and young person in Wales*’, one being *healthy, confident individuals who are ready to lead fulfilling lives as valued members of society.* Adequate health literacy plays an important role in achieving this purpose and is reflected within curriculum guidance spanning *functional* to *critical* domains, for example, *Have the skills and knowledge to manage everyday life as independently as they can.*

Another key development concerning health literacy is the renewed statutory focus on *Health and Well-being* as one of six distinct curriculum *areas of learning and experience*. Within each area, the curriculum framework is guided by *statements of what matters*. For *Health and Well-being* two of five include *Developing physical health and well-being has lifelong benefits* and *Our decision-making impacts on the quality of our lives and the lives of others.* Progression through the continuum of the *Health and Well-being* curriculum area is dependent on developing health literacy. Tracking measures of health literacy offers the potential to assess learner progression along their continuum of learning, and assess the impact of statutory focus on health and well-being.

A fundamental component of the *CfW* is a shift from a prescriptive national curriculum to one that offers autonomy to schools in local school-level curriculum design, reflecting the needs of its learners and wider community. The WHO advocate for co-designed approaches to health literacy (WHO, 2022), this is achievable in Wales with education a devolved responsibility and autonomous, school-level approaches to curriculum design. This enables schools to design curriculum areas aligned to their learners’ health and well-being needs (HAPPEN Wales, 2023), evidenced by national research platforms such as HAPPEN Wales (www.happen-wales.co.uk) currently engaging with over 500 primary schools across Wales. This offers opportunities for co-creation, co-production and scalable assessment of health literacy in Wales. However, to evaluate and track the impact of the *CfW*, measurement tools are required. These are currently lacking in Wales, though measures of wider child health and well-being exist including the HAPPEN Wales Survey. Urgent investment is needed for scalable measures of health literacy (WHO Regional Office for Europe, 2019).

This statutory focus on health and well-being through the *CfW* offers a unique opportunity to enhance the collective health literacy of current and future generations. Schools across Wales could be viewed as ‘*health literacy arenas*’, recognized by the WHO Regional Office for Europe as settings in which health literacy initiatives can be developed, carried out and evaluated. This reflects the concept of organizational health literacy, which in the context of schools refers to the behavioural (e.g. learning opportunities) and environmental measures (e.g. school framework or strategy) in place to strengthen health literacy (Kirchhoff and Okan, 2022). International case studies such as the HeLit-Schools project in Germany (Kirchhoff and Okan, 2022) offer learning opportunities for the *CfW*.

This focus on health literacy within the *CfW* is not restricted to impacting just learners in Wales. Health literacy is also a form of social and cultural capital (Kickbusch, 2008; WHO Regional Office for Europe, 2013b), individual capability fostered through the *CfW* can be dispersed and transferred to families and communities through the concept of distributed health literacy (McQueen *et al*., 2007). This can enhance the collective health literacy and capacity of the Welsh population and empower them as informed participants in decision-making about their health (Roundtable on Health Literacy; Board on Population Health and Public Health Practice; Institute of Medicine, 2013; WHO Regional Office for Europe, 2019). This also has implications for achieving other policy and strategy visions including those in *A healthier Wales* and *Prosperity for All.*

### Recent developments

In 2022, health literacy was identified as an Area of Research Interest (ARI) by the Senedd Cymru/Welsh Parliament’s Health and Social Care Committee to support work in line with the strategy for the Sixth Senedd (Senedd Cymru, 2021, 2022). *Increased levels of health literacy* is highlighted as a tool in achieving the strategy vision, through which *health inequalities and inequities* is a cross-cutting theme. At the time of writing, a review by the author has been presented to the Committee (Senedd Cymru, 2023). Embedding health literacy within emerging policy spanning health and social care, economy and education requires cross-governmental investment, support and ownership and collaboration with key stakeholders across research, policy and practice. This remains an open opportunity in Wales, and the development of a strategic action plan and task force group would facilitate and re-energize this.

## CALL FOR ACTION: WALES HEALTH LITERACY ACTION PLAN

Health literacy is a fundamental competency necessary to function within modern society. Increasing the health literacy capacity of the Welsh population offers one of the greatest potentials in reducing health inequalities and inequities, improving population outcomes and ensuring cost savings across the healthcare system. Wales has produced world-leading policy spanning health and social care, well-being, the economy and education. This offers opportunities to model and gain traction as a national-scale health literacy policy testbed. For children and young people, there are opportunities for co-production and tracking of health literacy within the *CfW* and its statutory focus on health and well-being as a curriculum area and overarching purpose, strengthening the collective health literacy of current and future generations.

As progress has stalled since 2010 and subsequent action plans, it is essential to re-energize health literacy as a national priority. This should be reflected as an explicit concept and goal within policy and strategy, inform the development of national action plans, supported by investment in monitoring, tracking and data infrastructure. This dearth of health literacy assessment across segments of society in Wales, particularly in children, limits the ability to capture the impacts of emerging policy and practice. However, Wales showcases world-leading data and data infrastructure, and opportunities for population data linkage of health, economic, education and administrative data exist through the SAIL Databank (Jones *et al.*, 2019). If maximized, Wales can position itself globally as a data-driven health literacy policy testbed (RMIT University, 2023). This can enable cross-national and international comparisons and provide insight and understanding into what interventions, policies and strategies are required and to who, tailor interventions to target populations and monitor and track the impact of these on individuals, communities and society.

This article proposes the following recommendations for consideration across research, policy and practice to build health literacy system capacity in Wales (Sørensen *et al*., 2021):

- ***Health-literate citizens***: clear, long-term, citizen-centred health and social care policy focus on co-developing responsible health-literate individuals in Wales.
- ***Health-literate systems***: strengthen research efforts, undertake robust and scalable research and capitalize population data linkage capabilities in Wales (e.g. SAIL Databank), enable national/international comparative data and insight.
- ***Health-literate policies***: explicit mention of health literacy within emerging policy and strategy, development of local/regional/national health literacy action plans, informed by the development of a health literacy strategic taskforce.
- ***Health-literate monitoring***: sustainable national assessment, monitoring and tracking of child health literacy and representation in the Global Atlas of Literacies of Health (RMIT University, 2023). This can position Wales globally, support health-literate systems in addition to:
  ◦ Assessment of learner progression within the *Health and Well-being* curriculum area;
  ◦ National impact evaluations of the *CfW* in enhancing health literacy;
  ◦ Understand children’s health literacy needs to inform the tailored design and evaluation of health literacy interventions at the local/regional/national level;
  ◦ Identify and inform policymaking priorities and assess the realization of wider policy visions.
